# Molecular Recognition by Templated Folding of an Intrinsically Disordered Protein

**DOI:** 10.1038/srep21994

**Published:** 2016-02-25

**Authors:** Angelo Toto, Carlo Camilloni, Rajanish Giri, Maurizio Brunori, Michele Vendruscolo, Stefano Gianni

**Affiliations:** 1 Istituto Pasteur – Fondazione Cenci Bolognetti and Istituto di Biologia e Patologia Molecolari del CNR, Dipartimento di Scienze Biochimiche “A. Rossi Fanelli”, Sapienza University of Rome, 00185 Rome, Italy; 2 Department of Chemistry, University of Cambridge, Cambridge CB2 1EW, UK

## Abstract

Intrinsically disordered proteins often become structured upon interacting with their partners. The mechanism of this ‘folding upon binding’ process, however, has not been fully characterised yet. Here we present a study of the folding of the intrinsically disordered transactivation domain of c-Myb (c-Myb) upon binding its partner KIX. By determining the structure of the folding transition state for the binding of wild-type and three mutational variants of KIX, we found a remarkable plasticity of the folding pathway of c-Myb. To explain this phenomenon, we show that the folding of c-Myb is templated by the structure of KIX. This adaptive folding behaviour, which occurs by heterogeneous nucleation, differs from the robust homogeneous nucleation typically observed for globular proteins. We suggest that this templated folding mechanism may enable intrinsically disordered proteins to achieve specific and reliable binding with multiple partners while avoiding aberrant interactions.

One of the most surprising recent findings in molecular biology is that as much as one third of the human proteome appears to be constituted by intrinsically disordered proteins (IDPs)^1^,^2^,^3^,^4^. These proteins play key roles in regulation and signalling, as their conformational heterogeneity enables them to act as network hubs by interacting with a range of different proteins and nucleic acids^5^,^6^,^7^,^8^,^9^,^10^. It is still unclear, however, how IDPs are capable of recognizing specifically their partners. Although it is widely believed that this type of molecular recognition process takes place by coupling folding and binding, detailed mechanistic studies to elucidate the molecular determinants of this reaction are still in their infancy and only relatively few mutational studies have been carried out to date to characterize the structural features of the corresponding transition states^11^,^12^,^13^,^14^,^15^,^16^,^17^.

A characteristic feature of small single-domain proteins is their ability to fold via an all-or-none reaction, with a main folding transition state (TS) being often the only experimentally detectable species between the native and denatured states^18^. Despite the inherent complexity of the protein folding process, which involves the concerted formation of hundreds of weak non-covalent interactions, the TS of globular proteins is often surprisingly robust and maintains its structural fingerprints when its stability is perturbed by mutagenesis or solvent variations^19^,^20^,^21^. Such robustness is a general feature arising from the presence of a well-defined folding nucleus, which confers to the transition state a native-like topology. This folding nucleus forms spontaneously under folding conditions (i.e. by homogeneous nucleation) and the overall structure of the TS resembles a distorted version of the native state^20^,^21^,^22^ with an average fraction of native-like contacts of about one third in nearly all globular proteins^23^.

To study the mechanism by which an IDP folds upon binding we investigated the transactivation domain of c-Myb (c-Myb), a 25-residue IDP that acquires an α-helical structure upon binding its partner KIX. KIX is a 88-residue globular domain of the CREB-binding protein (CBP), which is a co-activator that modulates the interaction between DNA-bound activator proteins and the components of the basal transcription complex^24^. KIX is the principal mediator of such interactions, via two distinct but coupled binding sites, named ‘c-Myb’ and ‘MLL’ sites, with reference to their specific ligands, the transactivation domain of c-Myb and the mixed lineage leukemia (MLL) protein^25^,^26^. We recently characterized the recognition mechanism for the binding of c-Myb and KIX^13^,^27^. The structure of c-Myb in complex with KIX is shown in Fig. 1. By analyzing the binding kinetics of KIX with a series of different variants of c-Myb, we showed that the mechanism involves a high geometrical precision^13^,^27^. This observation was mirrored by the very high content of native-like structure in the transition state, which exceeds what is generally observed in the folding reactions of globular proteins.

An interesting feature of the KIX domain is that its structure interconverts between a high-affinity state and a low-affinity state for c-Myb^28^, the equilibrium being controlled by a network of hydrophobic core residues that bridge the two binding sites^29^,^30^ (Fig. 1). The nature of the allosteric communication between the different sites has been investigated extensively, both computationally and experimentally^31^,^32^,^33^,^34^. In this work we determine the structure of the transition state for folding upon binding of c-Myb and establish the mechanism of the folding reaction induced by the binding to KIX. Our strategy is to design targeted perturbations of the interactions of the hydrophobic network of KIX using site-directed mutagenesis at positions I26, L43 and I72 (highlighted in Fig. 1), and then to challenge each of these variants with several variants of c-Myb, which were the same previously employed in binding to wild type KIX^13^. The use of experimental measurements as structural restraints in molecular dynamics simulations^22^ allows us to obtain at atomic resolution the structures of the four TSs corresponding to the binding between c-Myb versus KIX wild-type and its three mutational variants.

By analyzing the structures of these four TSs, we found a surprising variability of the folding pathway of c-Myb, which is templated by the structure of KIX and, in particular, by the exposure of hydrophobic surface in its binding site. This behaviour contrasts with what classically observed in the case of globular proteins and, we speculate, may represent a general feature of IDPs.

## Results and Discussion

### Effects of perturbing the hydrophobic network of KIX on the folding of c-Myb

**Figure 1:**
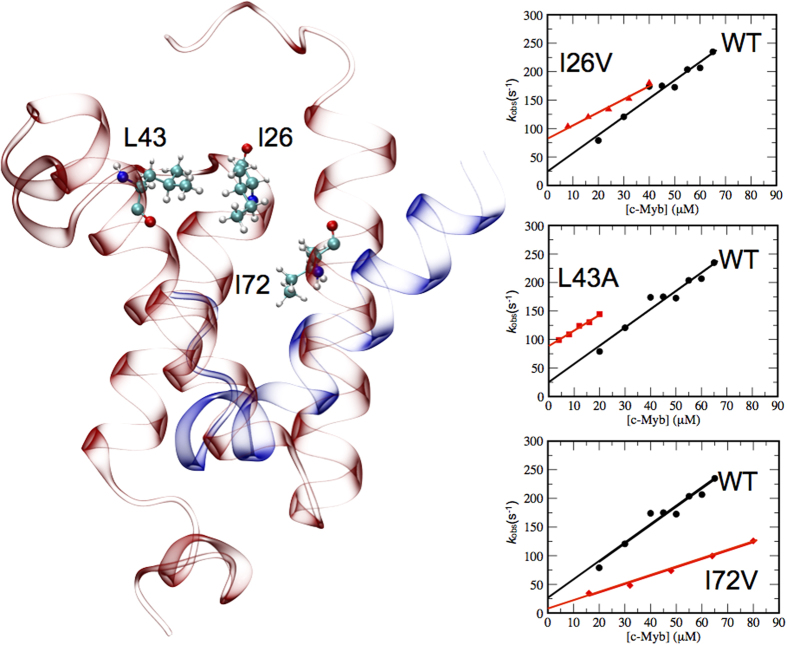
Hydrophobic network of KIX regulating c-Myb recognition. (Left) Structure of the complex between KIX (red) and c-Myb (blue), shown in cartoon (pdb: 1SB0). The key residues I26, L43 and I72 of KIX, previously identified as critical for ligand binding^29^,^30^, are highlighted in balls and sticks. (Right) Pseudo-first order kinetics for the binding of c-Myb to four variants of KIX, i.e. the wild type and three variants I26V, L43A and I72V. Rate constants were obtained by stopped-flow fluorimetry at a fixed concentration of KIX (typically ranging from 3 to 8 μM), mixed with increasing concentrations of c-Myb. All experiments were performed at 10 °C and the buffer used was 50 mM sodium phosphate, 150 mM KCl, 1 mM DTT, pH 7.2.

It has been recently suggested that a network of hydrophobic residues connecting the two active sites of KIX regulates the folding upon binding reaction of c-Myb^29^,^30^. In particular, positions I26, L43 and I72 play central roles in this hydrophobic network. These residues constitute part of a complex network that regulates the allosteric behaviour of the two binding sites of KIX^31^,^32^. In the present study, we targeted separately the three positions by producing three conservative site-directed variants, namely I26V, L43A and I72V, which were analysed by stopped-flow binding kinetics with c-Myb under pseudo-first-order conditions (Fig. 1). We observed detectable changes in association and dissociation rate constants, confirming that these residues regulate the affinity of KIX for its ligand. The values of the association and dissociation rate constants for the I26, L43 and I72 variants are reported in Table S1.

To test the effects of the allosteric core of KIX in governing the folding upon binding reaction of c-Myb, each of the variants (I26V, L43A and I72V) were challenged with different variants of c-Myb. We performed pseudo-first-order binding experiments using c-Myb and its site-directed mutants by mixing a constant concentration of KIX (3-8 μM) with increasing concentrations of c-Myb, typically ranging from 8 to 80 μM. The variants employed were the same as those reported in Table 1 of ref. 13. Under all conditions, the observed kinetics were consistent with a single-exponential behaviour, and the observed rate constants were found to follow simple bimolecular kinetics. The experimental data are reported in Figures S1–S3 and the kinetic parameters extracted from the data are listed in Table S1. For some variants of c-Myb reliable binding rate constants could not be measured; those positions are not reported in Table S1.

### Structural features of the transition states

A classic procedure to assess the structural features of a TS for folding is to perform a linear free energy relationship analysis (LFER), whereby changes in free energy of the TS are related to changes in equilibrium free energies^35^. The slope of the observed correlation, classically denoted as α, reflects the position of the transition state along the reaction coordinate. In the case of protein folding it has been suggested that the linearity of the LFER plot is distinctive of the so-called nucleation–condensation mechanism, whereby the transition state resembles a distorted version of the native state and the whole protein self-assembles around a weakly formed nucleus^20^,^21^. A comparative analysis of nearly all proteins reported to date revealed that the α-value in protein folding is robust and, for nearly all systems investigated, α is about 0.36^23^. Changes in α, typically consistent with a so-called Hammond behaviour^36^, may be observed only rarely and when mutants are considered at iso-stability. In a recent application of the LFER analysis to the recognition reaction of KIX and c-Myb, we showed that this IDP displays a linear LFER^13^. Unexpectedly, however, the calculated α-value of 0.89 suggested that the transition state displays a very high degree of native-like structure, being much more ordered than what typically observed for the folding transition state of globular proteins.

**Figure 2:**
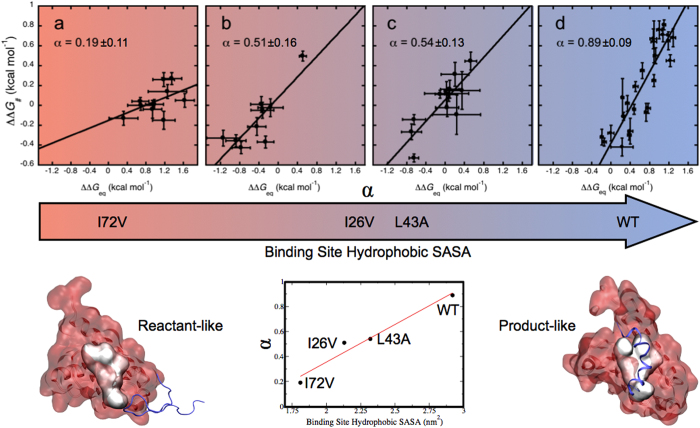
Unexpected variability of the KIX-c-Myb binding process. We used the LFER approach to analyse the binding of variants I26V, L43A and I72V of KIX to the different variants of c-Myb. (**a–d**) The slope obtained from a linear fit of each ΔΔG_eq_ vs ΔΔG^#^ plot yields the α value, reflecting the native-like structure observed in the reaction transition state and varying from 0 (reactant like) to 1 (product-like). Errors on α values have been estimated using a bootstrap analysis. The structure of the transition state for the reactant-like mutant I72V and for wild type KIX are shown for comparison. A scatter plot between the α value and the hydrophobic accessible surface area calculated using the hydrophobic residues in the binding site of each KIX variant is shown in the middle between the two structures. As described in the text, the observed linear correlation suggests the folding of c-Myb to occur via heterogeneous nucleation process templated by the structure of KIX.

To test the robustness of the structure of the transition state for the binding-induced folding reaction of c-Myb, we performed here a LFER analysis for the I26V, L43A and I72V variants (Fig. 2). We found that substitutions in the KIX domain have pronounced effects on the slope of the LFER plot, the α-value decreasing from 0.89 ± 0.09 for wild-type KIX, to 0.54 ± 0.13 for L43A, 0.51 ± 0.16 for I26V and 0.19 ± 0.11 for I72V. These results reflect a remarkable plasticity of the folding upon binding of c-Myb, which appears to be dictated by the structure of its partner KIX, and suggests an unexpected complexity of the underlying mechanism of molecular recognition. Furthermore, the analysis of the LFER plot for wild-type KIX (Fig. 2) highlights how the best fit of the data is shifted from the origin by a small but detectable value of about 0.5 kcal mol^−1^. This effect, which seems smaller in the case of the three mutants of KIX, may suggest the presence of additional steps in the mechanism of molecular recognition, which may be kinetically silent in binding experiments but yet detectable when the system is energetically perturbed (in this case by site-directed mutagenesis). This observation is consistent with recent findings by Peter Wright and co-workers^17^ suggesting that the binding-induced folding reaction of c-Myb occurs via a complex scenario involving features of both induced fit and conformational selection. Those results support our observation that the binding-induced folding mechanism of KIX is plastic and can be thus potentially modulated by mutagenesis.

### Determination of the transition state ensembles by Φ value analysis

The mutational analysis described above can be exploited to unveil the structural ensembles of the transition states of c-Myb corresponding to the wild type and the three mutational variants of KIX. Mutations that destabilize the transition state decreasing the association rate constant, target contacts that are involved in its structure^37^. Quantitatively, the strength of each contact is measured by the corresponding Φ value, which normalizes the stability loss of the transition state to that of the ground state. The reliability of Φ values has been extensively discussed in different studies, one of the main caveats being represented by changes in stability induced by a given substitution^23^,^37^,^38^,^39^,^40^,^41^. Therefore we excluded from the analysis the variants whose overall ΔΔG_eq_ was lower than 0.4 kcal mol^−1^, as customary^38^. The Φ value analysis has been successfully used in characterizing the binding induced folding of IDPs^12^,^13^,^14^,^15^,^16^,^17^,^42^. With the exception of the binding induced folding of the S-peptide to the S-protein, which is not a naturally occurring system, being obtained by the proteolytic digestion of ribonuclease A with subtilisin^43^, the IDPs studied so far did not show a relevant content of non-native interaction and thus ‘standard’ Φ values (i.e. between 0 and 1) have been typically observed.

**Figure 3:**
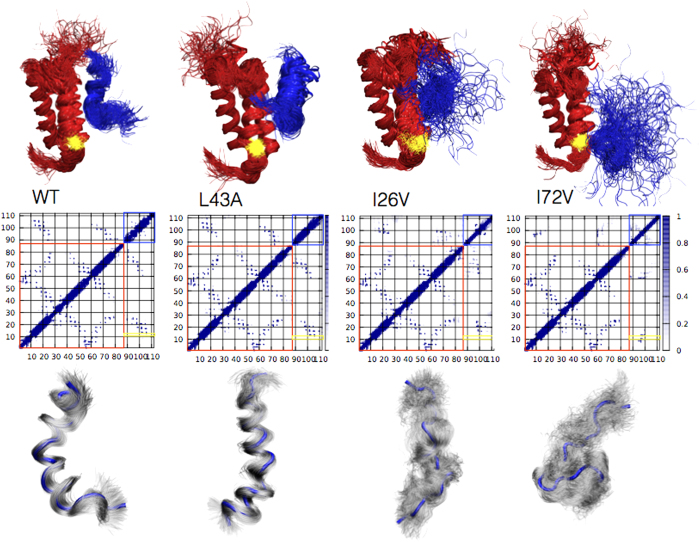
Transition state ensembles for c-Myb-KIX binding. The TS ensembles were obtained by using Φ-values as structural restraints in molecular dynamics simulations. The KIX domain is represented in red, whereas c-Myb is represented in blue. A contact map, highlighting the intra-molecular and inter-molecular contacts in each transition state, is reported in the middle (with a red and blue square identifying the KIX and c-Myb structures, respectively). Residue T11, which is critical in the I72V mutant but does not make any key contacts in wild type KIX, is highlighted in yellow both in the structures and in the contact maps. The structural ensemble of c-Myb in the transition state, together with its average structure (reported in blue), is shown below the relevant contact map.

The calculated Φ values obtained in this work for all the variants are reported in Table S1. This information can be used to obtain structural ensembles representing the transition states for the reaction^22^. In this approach, a trajectory is generated by integrating the equations of motion of a protein with a bias based on the incorporation of the Φ values in the force field. This methodology is analogous to the use of interatomic distances obtained through NOEs to determine native state structures by NMR spectroscopy^22^. The structures of the transition states for the folding of c-Myb upon binding wild-type KIX and its I26V, L43A and I72V variants, as obtained by restrained molecular dynamics simulations, are reported in Fig. 3.

Consistently with the LFER analysis (Fig. 2), a comparison of the variants of KIX reveals that the structure c-Myb is highly plastic in the transition state, with a progressive increase in structural disorder in the case of the variants (Fig. 3). We found that the variants I26V and L43A are more similar to the wild-type compared to the I72V mutant. The degree of native-like structure in the transition state of c-Myb, as probed by the α-value, is linearly correlated to the hydrophobic accessible surface area in the binding site of KIX (Fig. 2). Because the structure of c-Myb in the transition state is dictated by the conformation of KIX we propose that in the case of the intrinsically disordered protein that we studied here folding occurs upon binding via a heterogeneous mechanism, which we describe as ‘templated’ because the partner appears to participate directly in the nucleation process.

### Validation of the structures of the transition states

**Figure 4:**
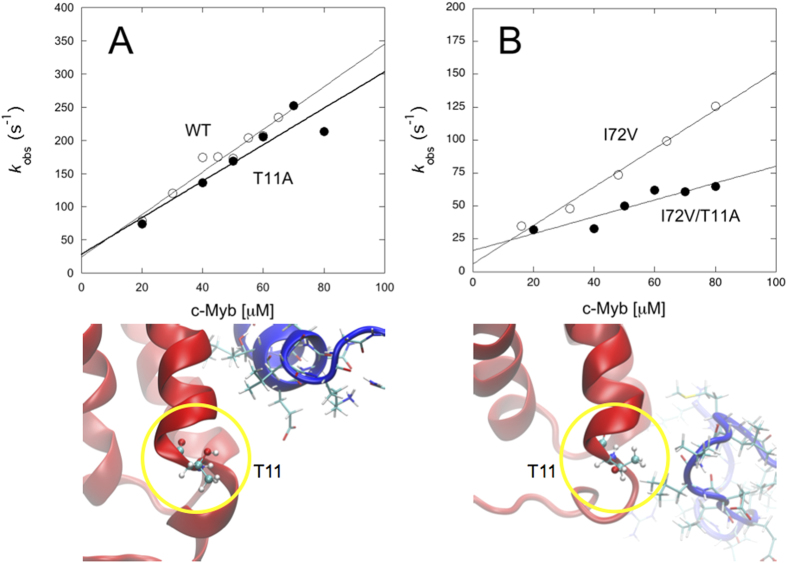
Validation of the transition state ensembles. To validate the transition state ensembles, we analysed the effects of the T11A substitution on the binding of c-Myb to wild-type KIX (left) and I72V (right). **(A)** Pseudo-first order binding kinetics of wild type KIX and T11A mutant versus wild type c-Myb. This substitution has a small effect on the binding kinetics, with association and dissociation rate constants equal to *k*_on_ = 2.7 ± 0.5 μM^−1^ s^−1^ and *k*_off_ = 28 ± 3 s^−1^ (wild type values *k*_on_ = 3.2 ± 0.3 μM^−1^ s^−1^ and *k*_off_ = 25 ± 3 s^−1^). **(B)** Pseudo-first order binding kinetics of I72V and I72V/T11A versus wild type c-Myb. In this case, substitution in position 11 (highlighted in yellow in Fig. 3) has a detectable effect, the calculated rate constants being *k*_on_ = 0.6 ± 0.1 μM^−1^ s^−1^ and *k*_off_ = 16 ± 2 s^−1^ for I72V/T11A, and *k*_on_ = 1.46 ± 0.09 μM^−1^ s^−1^ and *k*_off_ = 6 ± 1 s^−1^ for I72V. The structure of the transition states for the KIX wild type and I72V, highlighting the conformation of T11 in the two cases, are shown below each graph.

A comparison of the structures of the four different transition states (Fig. 3) suggests that the substitution I72V induces a structural change of the binding pocket, with clear non-native interactions at the interface between the two proteins. Within this context, the residue T11 of KIX (highlighted in yellow in Figs 3 and 4) is particularly relevant being located at the centre of the cluster of non-native interactions in the TS of the I72V, though not engaged in any detectable contacts in the case of wild-type KIX. Thus, to provide an experimental validation of the structure of the transition states described above, we designed a variant of c-Myb by mutating position T11. Fig. 4 shows the effects of the T11A substitution on the pseudo-first-order kinetics of the binding of c-Myb the wild-type and I72V variants of KIX. As expected, we found that the T11A substitution had little effect on both the association and dissociation rate constants in the case of wild-type KIX, while affecting both rate constants in the case of the I72V variant. This finding confirms that the I72V substitution perturbs the mechanism of binding of KIX to c-Myb and determines a shift of the binding site, which in I72V clearly involves position T11.

### Molecular recognition by templated folding

Previous computational and experimental work investigated the complex behaviour of KIX as well as its allosteric features^31^,^32^,^33^,^34^. The malleability of the folding of c-Myb upon binding KIX indicates that c-Myb adapts its folding process depending on the structure of its binding partner. To further characterise this process we analyzed the relationships between the structure and the thermodynamics of the transition states, by performing a cross-correlation analysis in which we systematically compared the different parameters. Inspection of Fig. 3 indicates that one of the main effects of the substitutions of KIX on the structure of the transition state of c-Myb is to increase the heterogeneity of the conformational ensemble, which appears rather ordered when binding wild-type KIX whereas is very heterogeneous when binding the mutant I72V, with the variants I26V and L43A being very similar and displaying an intermediate behaviour. The degree of native like-structure in the TS of c-Myb is thus very malleable and dictated by the solvent accessible hydrophobic surface area in the binding site of KIX (Fig. 2), which can be tuned by mutagenesis as shown above.

**Figure 5:**
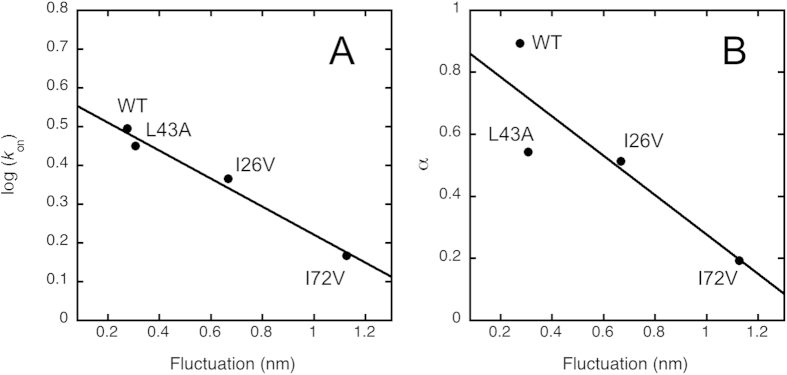
Mechanistic basis of the templated folding process. To identify the mechanistic basis of the templated folding process we carried out a cross correlation analysis of the c-Myb to KIX binding reaction. The two panels show the correlation between the fluctuation of the transition state ensemble around the average structure with (**A**) the logarithm of the association rate constant, and (**B**) the α value obtained from LFER analysis.

To better quantify the degree of conformational heterogeneity of the transition states, we computed the average deviation from the mean structure, which relates to the overall fluctuation of the ensembles. This analysis resulted in values of 0.28 nm, 0.31 nm, 0.67 nm and 1.13 nm for the wild-type, L43A, I26V and I72V variants, respectively. The correlations between these values and: (i) the logarithm of the association rate constants and (ii) their respective α values are shown in Fig. 5. The satisfactory linear correlations provide a mechanistic basis for the templated folding process, with the I72V mutant displaying the lowest association rate constant and the highest degree of structural heterogeneity. Moreover, the good correlation between the fluctuations around the structure of the transition state and α values (Fig. 5) confirms that the LFER analysis and the restrained molecular dynamics simulations are both valuable methods to characterise the structural properties of the transition states.

## Conclusions

**Figure 6:**
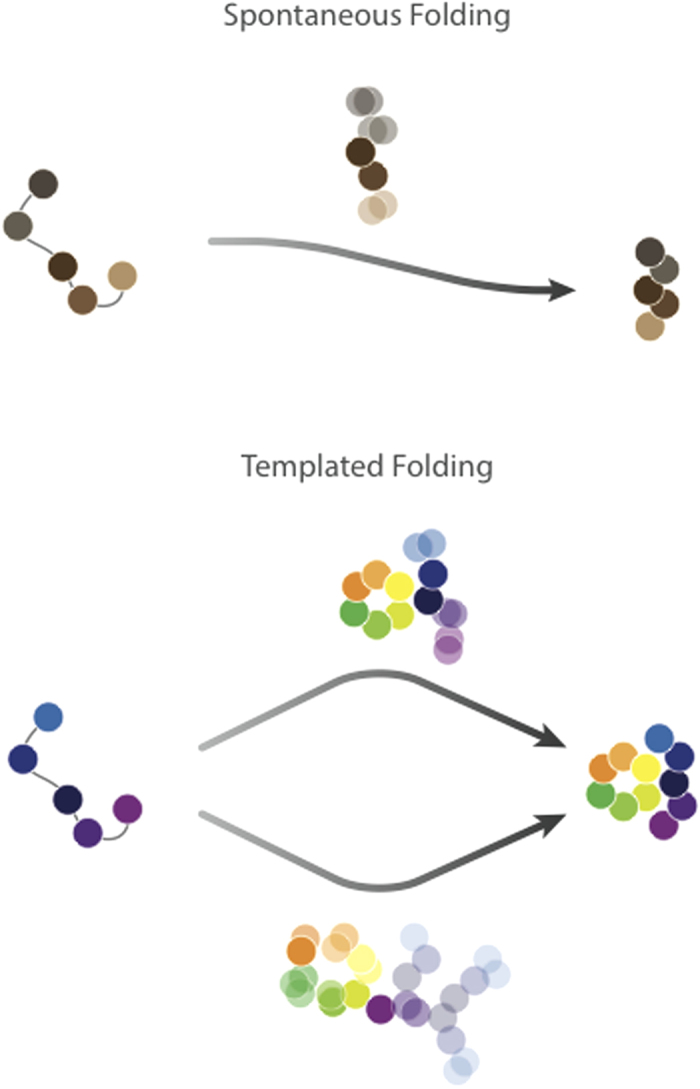
Schematic illustration of the templated folding mechanism. The analysis of the thermodynamic and structural features of the folding upon binding mechanism of c-Myb reveals that this IDP folds via a templated mechanisms, which involves a heterogeneous nucleation process induced by the structural features of the ligand. This view implies a structural malleability of the transition state, which contrasts the robustness typically observed in the folding pathway of globular proteins, where the transition state forms by a spontaneous nucleation process (homogeneous nucleation) and is a distorted version of the native state with a highly conserved fraction of native-like contacts^19^,^21^,^23^.

We have shown that c-Myb folds in a templated manner upon binding its partner KIX. Our results illustrate how the structure of KIX dictates the transition state of folding of c-Myb, which is clearly changed even by substitutions on KIX not directly involving the binding site. In fact, this type of ‘templated folding’ behaviour, summarized graphically in Fig. 6, is in contrast with the classic folding typically observed for globular proteins, which tends to be robust and governed by the topology of the native states. These observations, which complement the recent experimental and computational studies by Brooks, Mapp, Clarke and co-workers^31^,^32^,^33^, allow the folding-upon-binding reactions induced by KIX to be described in great detail.

We suggest that the propensity of an IDP to fold by being templated by the structure of its partner may represent a general mechanism whereby multiple partners can be specifically recognized. This mechanism confers robustness to the molecular recognition process and minimizes the possibility of forming aberrant interactions with potential pathological consequences.

## Materials and Methods

### Site-directed mutagenesis and protein purification

The variants of KIX named I26V, L43A, I72V, and T11A, and all the variants of c-Myb were obtained by using the Quik-Change Mutagenesis kit (Stratagene) according to the manufacturer’s instructions, and the substitutions were confirmed by DNA sequencing. Template DNA used for KIX mutants was KIX Y73W. Expression and purification, for KIX and c-Myb variants, were performed as described previously^13^,^27^.

### Stopped-flow measurements

Binding kinetics experiments were carried out on a single-mixing SX-18 stopped-flow instrument (Applied Photophysics); the excitation wavelength used was 280 nm and the fluorescence emission was collected using a 320-nm–cutoff glass filter. Pseudo-first order binding experiments were performed mixing a constant concentration of each of the KIX variants, 3 μM or 8 μM, versus increasing concentrations of all the c-Myb mutants, ranging between 8 μM and 80 μM. Because of the difference in concentrations between KIX and c-Myb, the pseudo-first order condition could be approximated in nearly all the experiments preformed. For all binding experiments the temperature was 10 °C and the buffer used was 50 mM sodium phosphate, 150 mM KCl, 1 mM DTT, pH 7.2. All reagents were of analytical grade. All the experiments were repeated 3–6 times and the average values with their associated standard deviations are reported in Supplemental Figures S1–S3.

In all cases the association (*k*_on_) and dissociation (*k*_off_) rate constants were determined by linear regression using the following equation:


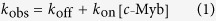


where *k*_obs_ is the observed binding rate constants. Because of the relatively high values of *k*_off_ for the variants of c-Myb, the analysis of the dissociation rate constants was performed by linear extrapolation.

### Determination of the transition state ensembles

Binding transition state ensembles were determined following a standard procedure based on the interpretation of Φ-value analysis in terms of fraction of native contacts. Briefly, given a set of experimental Φ-values, a pseudo energy term has been added to a force field as the squared difference between experimental and simulated Φ-values in order to maximize the agreement with the experimental value while keeping the simulation stable. The Φ-value for a residue is calculated from the fraction of native contacts that it makes in a conformation. Given two residues that are not nearest neighbours, the native contacts between them are defined as the number of heavy side-chain atoms within 0.65 nm in the native structure. As a control, an additional simulation of the WT transition state ensemble has been performed using only the Φ-values for residues 1, 3, 18 and 22. The resulting ensemble is consistent with the ensemble obtained with all the data but slightly more native like, indicating that the calculated is robust with respect to the number of mutants taken into account.

The different transition state ensembles were generated using simulated annealing. Each ensemble is the results of 300 annealing cycles, 150 ps long, in which the temperature is varied between 283 K and 383 K. Molecular dynamics simulations were performed using the Amber03W force field with the TIP4P05 water model. All the simulations were run in GROMACS using PLUMED2^44^. The van der Waals and Coulomb interactions were implemented with a cutoff at 0.9 nm, and long-range electrostatic effects were treated with the particle mesh Ewald method on a grid with a mesh of 0.1 nm. All simulations were carried out in the canonical ensemble at constant volume and by thermosetting the system using a stochastic velocity rescaling^45^. The starting conformation was taken from an available NMR structure (PDB code 1SB0) and substitutions were obtained using Scwrl4. Each structure was solvated in ~7,000 water molecules. A standard 283 K simulation, 100 ns long, was performed as a reference for the native state ensemble of each mutant (wild-type, I26V, L43A and I72V). From the resulting ensembles the hydrophobic exposed solvent accessible surface area (SASA) has been calculated as the sum of the SASA of the residues L14, L18, L22, A25, L68 and I/V72 using the GROMACS g_sas tool.

## Additional Information

**How to cite this article**: Toto, A. *et al.* Molecular Recognition by Templated Folding of an Intrinsically Disordered Protein. *Sci. Rep.* **6**, 21994; doi: 10.1038/srep21994 (2016).

## Supplementary Material

Supplementary Information
