# Supplementary material for: Molecular Recognition by Templated Folding of an Intrinsically Disordered Protein
Source: Sci Rep. 2016 Feb 25;6:21994. doi: 10.1038/srep21994 (PMC4766501; doi:10.1038/srep21994)
Supplement: Supplementary Information [file srep21994-s1.pdf]

Supporting Information File

Molecular Recognition by Templated Folding of an Intrinsically Disordered Protein

Angelo Toto[1,a], Carlo Camilloni[2,a] , Rajanish Giri[1,*], Maurizio Brunori[1], Michele Vendruscolo[2,§] and Stefano Gianni[1,2,§]

[1]*Istituto Pasteur – Fondazione Cenci Bolognetti and Istituto di Biologia e Patologia Molecolari del CNR, Dipartimento di Scienze Biochimiche "A. Rossi Fanelli" Sapienza, University of Rome, 00185 Rome, Italy.*

[2]*Department of Chemistry, University of Cambridge, Cambridge CB2 1EW, UK*

Table S1. Binding parameters to KIX variants for binding to c-Myb and its site directed mutants.

I26V						
Protein	k_{on} μM^{-1} s^{-1}	k_{off} s^{-1}	K_D μM	$\Delta\Delta G_{\#}$ kcal mol^{-1}	$\Delta\Delta G_{eq}$ kcal mol^{-1}	Φ
WT	2.3 ± 0.19	83 ± 5	36 ± 4			
E2A	4.3 ± 0.33	33 ± 9	7.6 ± 2.1	-0.36 ± 0.06	-0.87 ± 0.21	0.41 ± 0.11
K3A	2.3 ± 0.27	44 ± 7	19 ± 3.9	0.01 ± 0.08	-0.35 ± 0.22	- [a]
K6A	2.5 ± 0.34	50 ± 9	20 ± 4.5	-0.05 ± 0.08	-0.33 ± 0.24	- [a]
L11A	2.4 ± 0.38	65 ± 10	27 ± 6.0	-0.02 ± 0.09	-0.16 ± 0.26	- [a]
S14A	0.96 ± 0.06	85 ± 4	90 ± 5.5	0.50 ± 0.05	0.51 ± 0.07	0.97 ± 0.12
T15S	4.1 ± 0.49	20 ± 5	4.7 ± 1.1	-0.33 ± 0.08	-1.14 ± 0.35	0.29 ± 0.10
L19A	4.4 ± 0.35	100 ± 9	22.4 ± 2.7	-0.37 ± 0.06	-0.26 ± 0.17	-
Q22A	4.9 ± 0.34	45 ± 9	9.1 ± 1.9	-0.44 ± 0.06	-0.77 ± 0.19	0.56 ± 0.15
Q23A	3.4 ± 0.34	55 ± 8	16.2 ± 6.8	-0.21 ± 0.09	-0.44 ± 0.19	0.48 ± 0.16
L43A						
Protein	k_{on} μM^{-1} s^{-1}	k_{off} s^{-1}	K_D μM	$\Delta\Delta G_{\#}$ kcal mol^{-1}	$\Delta\Delta G_{eq}$ kcal mol^{-1}	Φ
Wt	2.8 ± 0.17	88 ± 2	31 ± 2.0			
K1A	4.4 ± 0.72	41 ± 10	9 ± 2.6	-0.27 ± 0.08	-0.70 ± 0.21	0.37 ± 0.16
E2A	3.5 ± 0.40	35 ± 11	10 ± 3.2	-0.13 ± 0.05	-0.65 ± 0.24	0.20 ± 0.11
K3A	2.3 ± 0.10	59 ± 3	26 ± 1.6	0.11 ± 0.04	-0.11 ± 0.10	- [a]
K6A	3 ± 1.2	160 ± 30	50 ± 19	-0.09 ± 0.20	0.24 ± 0.50	- [a]
L11A	2.1 ± 0.31	79 ± 4	37 ± 5.8	0.16 ± 0.09	0.10 ± 0.19	- [a]
S14A	1.6 ± 0.32	72 ± 9	45 ± 10	0.31 ± 0.12	0.20 ± 0.29	- [a]
T15S	2.1 ± 0.50	120 ± 19	58 ± 16	0.15 ± 0.19	0.34 ± 0.37	- [a]
E16A	2.3 ± 0.27	83 ± 8	36 ± 5.4	0.11 ± 0.07	0.07 ± 0.19	- [a]
N17A	2.3 ± 0.75	80 ± 20	33 ± 14	0.11 ± 0.12	0.03 ± 0.46	- [a]
L19A	7.2 ± 0.42	72 ± 6	9.9 ± 1.0	-0.53 ±	-0.65 ±	0.82 ±

				0.04	0.10	0.14
Q22A	1.3 ± 0.43	110 ± 12	80 ± 7.7	0.43 ± 0.09	0.53 ± 0.12	0.80 ± 0.14
Q23A	2.9 ± 0.47	100 ± 12	34 ± 6.9	-0.02 ± 0.09	0.04 ± 0.24	- [a]
I72V						
Protein	k_{on} μM^{-1} s^{-1}	k_{off} s^{-1}	K_D μM	$\Delta\Delta G_{\#}$ kcal mol^{-1}	$\Delta\Delta G_{eq}$ kcal mol^{-1}	Φ
WT	1.46 ± 0.09	6 ± 1	4.2 ± 0.8			
K1A	1.15 ± 0.12	46 ± 7	40 ± 7.2	0.14 ± 0.07	1.26 ± 0.22	0.11 ± 0.04
K3A	0.9 ± 0.11	32 ± 6	35 ± 7.7	0.26 ± 0.07	1.18 ± 0.23	0.22 ± 0.08
I5A	1.4 ± 0.10	35 ± 5	24 ± 4.0	0.01 ± 0.05	0.98 ± 0.19	0.01 ± 0.01
K6A	0.92 ± 0.09	43 ± 5	47 ± 7.3	0.27 ± 0.06	1.35 ± 0.21	0.20 ± 0.06
E7A	1.46 ± 0.04	21 ± 2	14.4 ± 1.4	0.00 ± 0.05	0.69 ± 0.28	0.00 ± 0.02
L11A	1.6 ± 0.10	36 ± 5	23 ± 3.7	-0.04 ± 0.09	0.94 ± 0.20	-0.05 ± 0.02
E16A	1.9 ± 0.27	66 ± 12	35 ± 8.0	-0.15 ± 0.09	1.18 ± 0.24	-0.12 ± 0.05
L19A	1.3 ± 0.15	103 ± 8	77 ± 11	0.05 ± 0.07	1.63 ± 0.20	0.03 ± 0.01
Q22A	1.37 ± 0.06	20 ± 3	14 ± 2.4	0.04 ± 0.04	0.68 ± 0.18	0.05 ± 0.03
Q23A	1.8 ± 0.20	14 ± 6	7.6 ± 3.9	-0.13 ± 0.07	0.32 ± 0.31	- [a]

[a] These mutants display a $\Delta\Delta G_{eq} < 0.4$ kcal mol^{-1}, which prevents accurate calculation of Φ-values.

All the mutants of c-Myb reported in reference (12) were mixed against both I26V, L43A, I72V. However, in the case of some mutants, we could not measure a reliable binding transition on the millisecond time scale. Values reported in the Table refer to the mutants that resulted in a measurable transition using a stopped-flow apparatus.

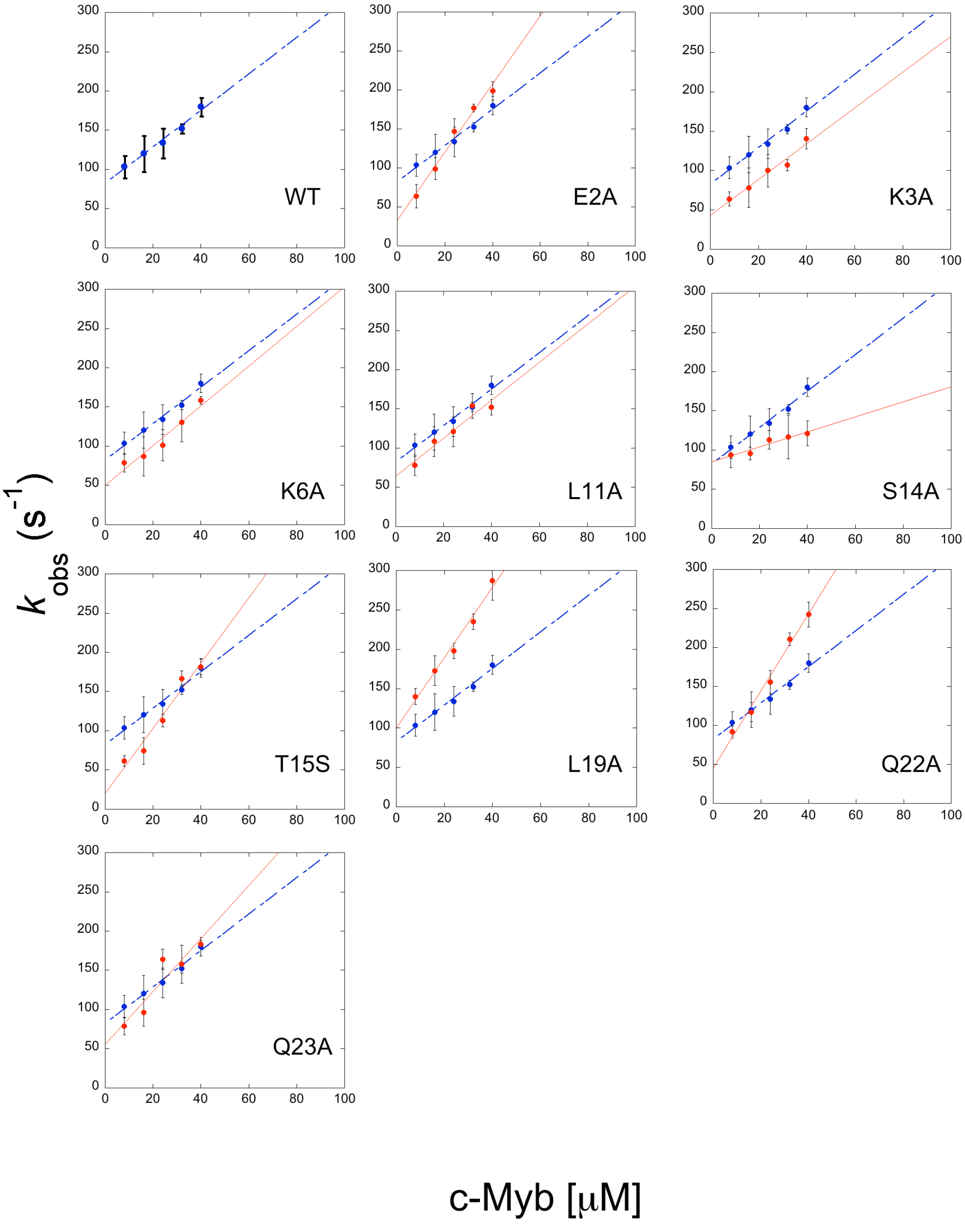

Figure S1. Pseudo–first-order kinetics of the binding between KIX I26V and the different c-Myb mutants at pH 7.2 and 10 °C. Data were recorded at a constant concentration of KIX, typically between 3 and 8 μM, mixed with variable concentrations of c-Myb and its site-directed mutants. The linear concentration dependence for wild-type c-Myb is shown for comparison throughout, depicted in blue dots and broken blue lines. Observed time courses were consistent with single-exponential behavior in all cases. Each data point refers to the average of 3 to 6 individual experiments. The calculated standard deviation of each data point is reported as an error bar in the plots.

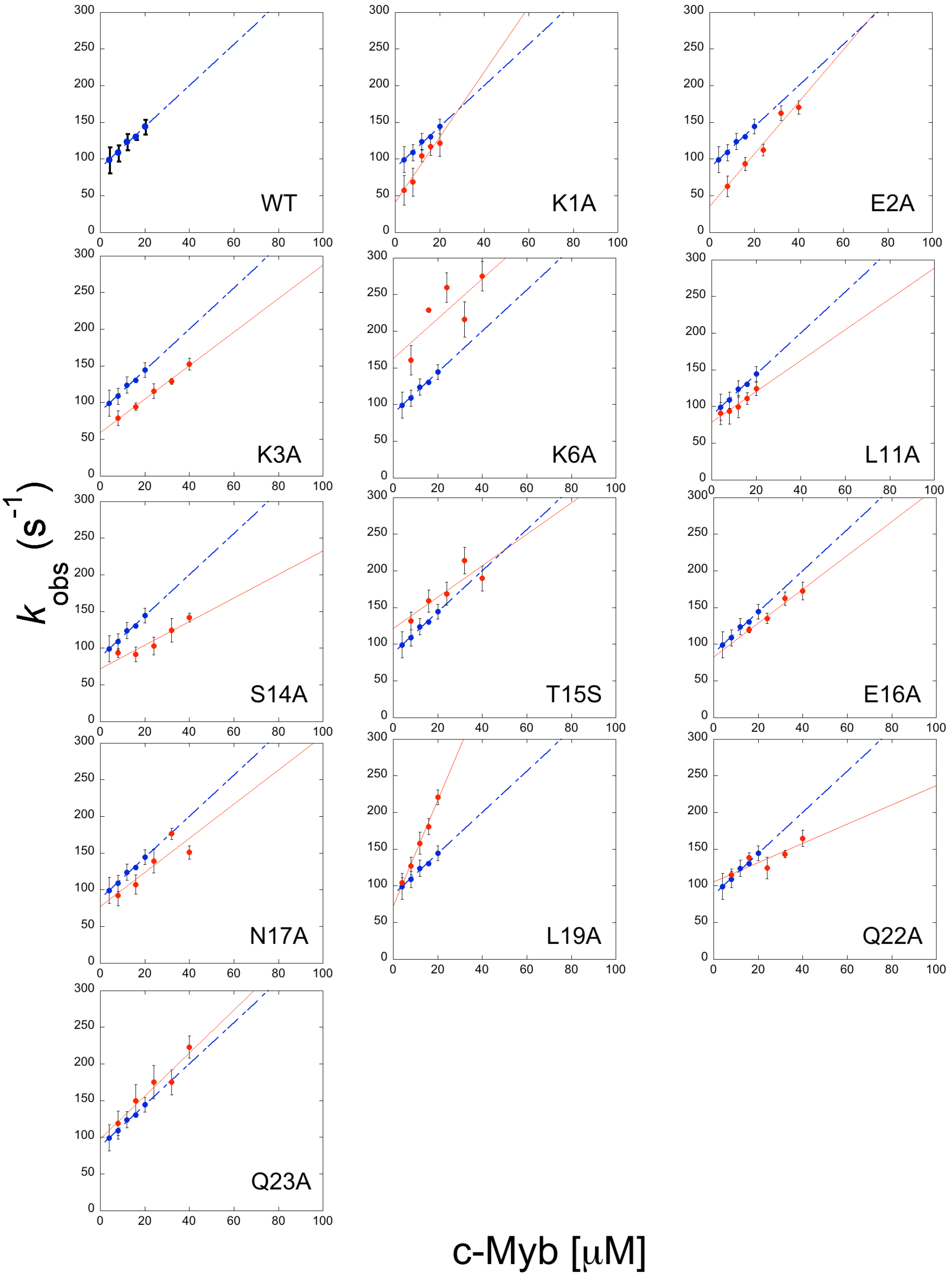

Figure S2. Pseudo–first-order kinetics of the binding between KIX L43A and the different c-Myb mutants at pH 7.2 and 10 °C. Data were recorded at a constant concentration of KIX, typically between 3 and 8 μM, mixed with variable concentrations of c-Myb and its site-directed mutants. The linear concentration dependence for wild-type c-Myb is shown for comparison throughout, depicted in blue dots and broken blue lines. Observed time courses were consistent with single-exponential behavior in all cases. Each data point refers to the average of 3 to 6 individual experiments. The calculated standard deviation of each data point is reported as an error bar in the plots.

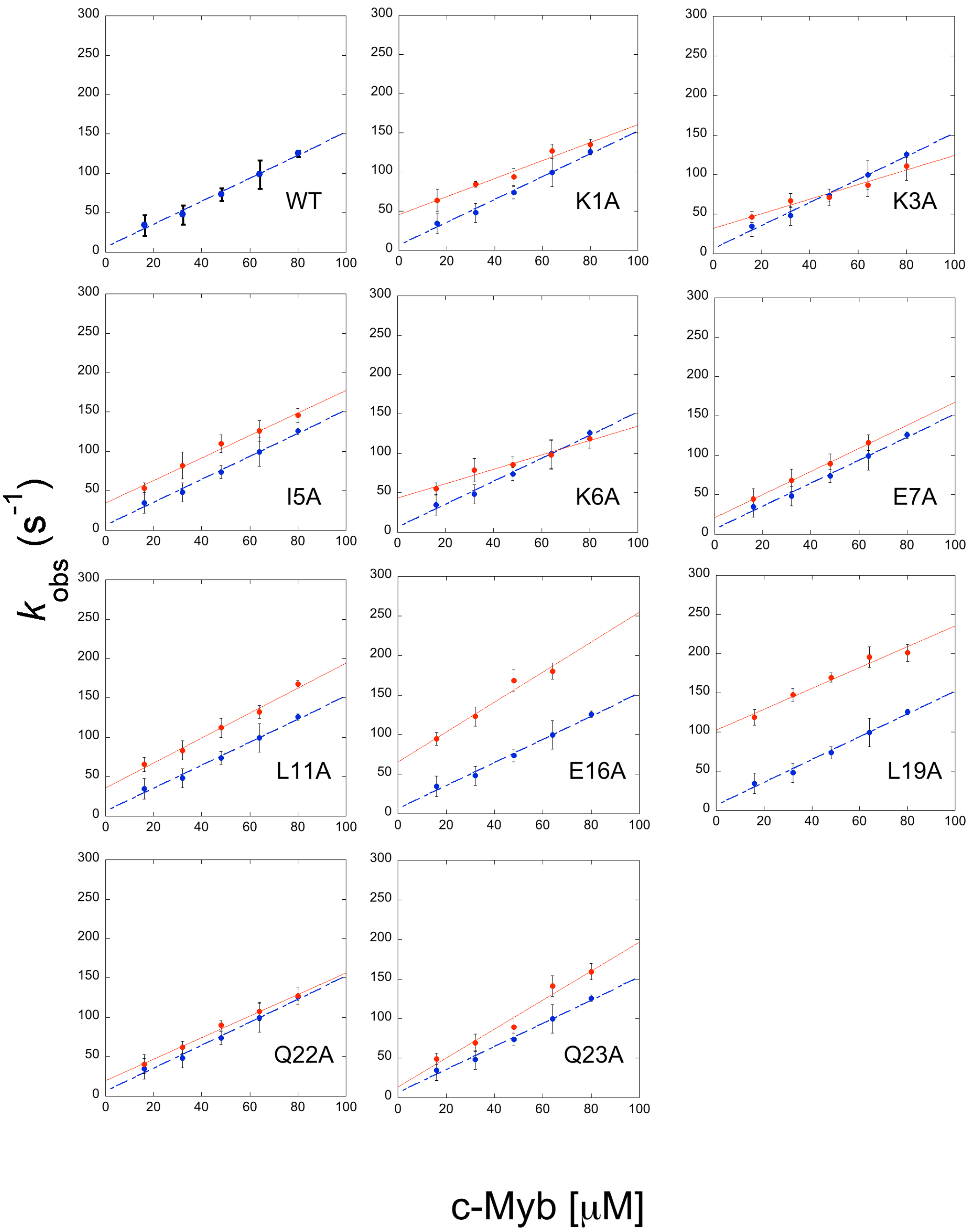

Figure S3. Pseudo–first-order kinetics of the binding between KIX I72V and the different c-Myb mutants at pH 7.2 and 10 °C. Data were recorded at a constant concentration of KIX, typically between 3 and 8 μM, mixed with variable concentrations of c-Myb and its site-directed mutants. The linear concentration dependence for wild-type c-Myb is shown for comparison throughout, depicted in blue dots and broken blue lines. Observed time courses were consistent with single-exponential behavior in all cases. Each data point refers to the average of 3 to 6 individual experiments. The calculated standard deviation of each data point is reported as an error bar in the plots.
